# Multifunctional Silk and Gelatin Composed Microneedle Patches for Enhanced Wound Healing

**DOI:** 10.1002/smmd.137

**Published:** 2025-02-26

**Authors:** Lu Fan, Li Wang, Xiaoju Wang, Minli Li, Hongcheng Gu, Hongbo Zhang

**Affiliations:** ^1^ Joint Centre of Translational Medicine, Wenzhou Key Laboratory of Interdiscipline and Translational Medicine The First Affiliated Hospital of Wenzhou Medical University Wenzhou China; ^2^ Pharmaceutical Sciences Laboratory Åbo Akademi University Turku Finland; ^3^ State Key Laboratory of Bioelectronics School of Biological Science and Medical Engineering Southeast University Nanjing China; ^4^ Turku Bioscience Centre University of Turku and Åbo Akademi University Turku Finland

**Keywords:** antimicrobial, hydrogel, microneedle, multifunctional, wound healing

## Abstract

Wound healing has been a continuous critical focus in clinical practice, posing the ongoing challenges and burdens to patients. Current attempts tend to develop multi‐drug loaded patches with spatial design. Herein, we present a multifunctional microneedle patch that integrates different drugs into separated regions for wound treatment. The microneedle patch is composed of silk fibroin‐methacryloyl (SilMA) as the base, loaded with silver nanoparticles (AgNPs) and has gelatin methacryloyl (GelMA) tips loaded with vascular endothelial growth factor (VEGF). The backing is endowed with antimicrobial properties by AgNPs act as an antimicrobial barrier against bacterium invasion. In addition, the tips encapsulated with VEGF can effectively promote cell proliferation and angiogenesis, which is favorable for wound repair. Based on these characteristics, such an integrated microneedle system significantly prevented bacterial infection and promoted wound healing in vivo. Therefore, it is conceived that such a system can find more practical values in wound healing and other fields.

## Graphical abstract

A multifunctional microneedle patch that integrates different drugs into separated regions for wound treatment is presented. Such patch has outstanding antimicrobial properties, as well as effective cell proliferation and angiogenesis ability, favorable for wound repair.

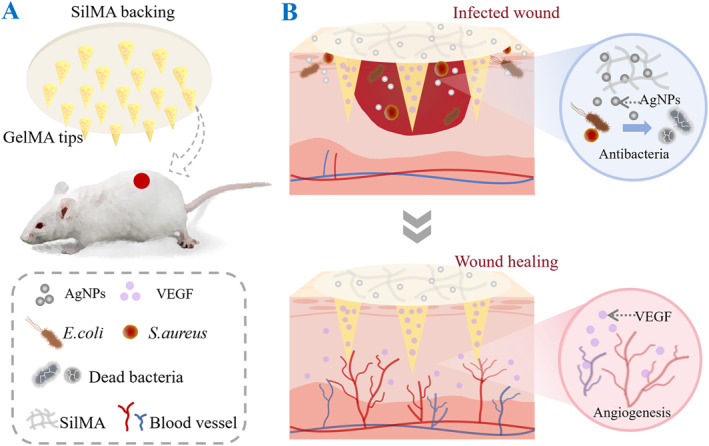

**Summary**

- Hydrogel microneedle patch composed of SilMA basement and GelMA tips.
- The microneedle patch is designed to integrate antibacterial agents and growth factors into separated regions.
- The microneedle patch provides multifunction for wound healing.

## 1 Introduction

The skin serves as the body’s first line of defense, playing a crucial role in protecting the body from harmful external factors [1, 2]. When it is damaged, the skin becomes susceptible to infections, inflammation, and other complications [3, 4, 5]. Thus, efficient wound healing is extremely vital for restoring the skin barrier function [6, 7]. With this purpose, various patches loaded with functional drugs have been explored, such as antibacterial agents and growth factors [8, 9, 10, 11, 12]. For example, antibacterial agents are highly effective in combating microbes and against bacterium invasion, aiming at the surface layer of susceptible wounds [13, 14, 15]. Growth factors have the ability to promote endothelial cell proliferation and migration, thus driving angiogenesis, which plays a critical role in the tissue regeneration phase [16, 17, 18, 19]. While, attributed to their different targeting wound sites and the varying stages of healing process [20, 21], the combination profiles of antibacterial agents and growth factors are still facing challenges [22, 23, 24]. Meanwhile, the direct mixing of different drugs in one material easily leads to potential interference between the drugs, also resulting in unsatisfactory therapeutic outcomes [25, 26, 27]. Thus, a patch with a spatial design to load antibacterial agents and growth factor separately, allowing each drug to exert optimal efficacy, is highly desired.

**FIGURE 1:**
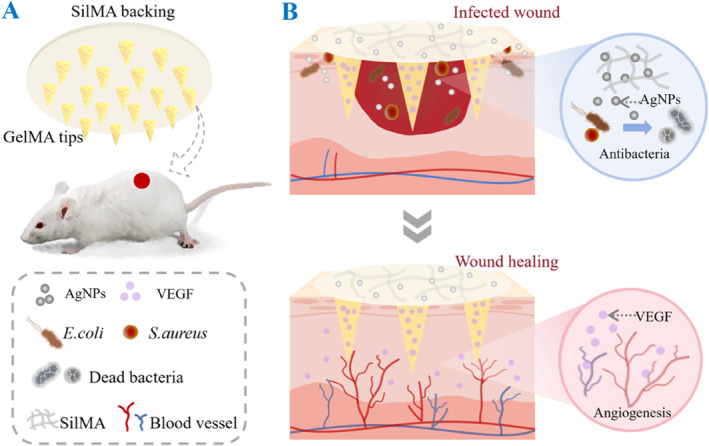
Schematic illustration of (A) the multifunctional microneedle patch and (B) its application on wound healing.

In this paper, we proposed a multifunctional microneedle patch designed to integrate distinct drugs into separated regions for wound healing treatments, as schemed in Figure 1. A microneedle patch with a spatial structure is known as a painless and portable device [28, 29, 30, 31, 32, 33]. Our microneedle patch is composed of gelatin methacryloyl (GelMA) needle tips and a silk fibroin‐methacryloyl (SilMA) backing base. The tips could penetrate the stratum corneum and deliver drugs into the subcutaneous layer, whereas the backing layer acts as a dressing to first cover the wound. Additionally, silver nanoparticles (AgNPs) with an efficient antimicrobial effect were incorporated into the backing layer. This backing was endowed with antimicrobial properties and acted as an antimicrobial barrier that effectively prevented wound infection. Moreover, vascular endothelial growth factor (VEGF) was encapsulated within the GelMA tips, which could effectively promote cell proliferation and angiogenesis, thus accelerating tissue regeneration and repair. Furthermore, in vivo experiments demonstrated that such a microneedle system significantly promoted wound and tissue healing. Benefiting from these advantages, we believe that the multifunctional microneedle system will have promising potential for broader applications in wound healing.

## 2 Results and Discussion

**FIGURE 2:**
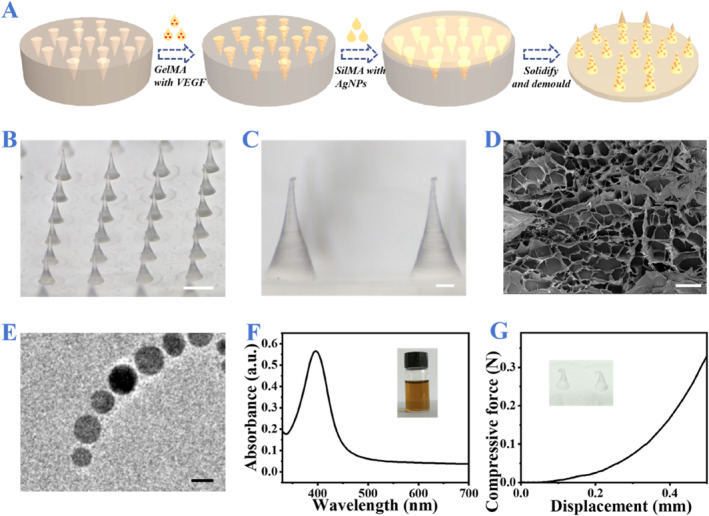
(A) Scheme of fabrication of MMN patches. (B) Optical image of the MMN patch. Scale bar: 500 μm. (C) Local enlarged image of MMN patch. Scale bar: 100 μm. (D) SEM image of the SilMA baking of MMN patch. Scale bar: 50 μm. (E) TEM image and (F) UV‐vis spectra of AgNPs. Scale bar in (E): 20 nm. (G) Compressive force‐displacement curve of the per tip of MMN patch.

In a typical experiment, the fabrication of the multifunctional microneedle (MMN) patches was produced by template replication. As shown in Figure 2A, gelatin methacryloyl (GelMA) solution mixed with VEGF was introduced into the tips of the negative mold. Afterward, silk fibroin‐methacryloyl (SilMA) solution carrying Ag nanoparticles (AgNPs) was added to the backing part of the mold. After UV irradiation, the tips and backing parts were totally cross‐linked and solidified. Finally, the entire microneedle patch was carefully demoulded. The obtained MMN patch displayed a desirable morphology, whose tips had uniformly conical shapes and neatly distributed on the backing base (Figure 2B,C). Besides, the tip height was measured as about 650 μm (Supporting Information S1: Figure S1). Furthermore, scanning electron microscopy (SEM) was used to characterize the morphoy and structure of the MMN patch. The results showed that the MMN patches featured completely conical‐shaped tips and a porous SilMA backing structure favorable for wound breathability (Supporting Information S1: Figure S2 and Figure 2D). Besides, the synthesized AgNPs were characterized by a transmission electron microscope (TEM), UV‐Visible absorption spectroscopy and dynamic light scattering (DLS). As shown in Figure 2E,F and Supporting Information S1: Figure S3, AgNPs exhibited a spherical shape, a relatively uniform size distribution and stable dispersion, suggesting their usability in the following applications. In addition, the compressive force of the MMNs was monitored and the tolerable limit of per needle was determined to be 0.33 N (Figure 2G). Taken together, such MMN patches exhibited excellent morphology and mechanical performance.

**FIGURE 3:**
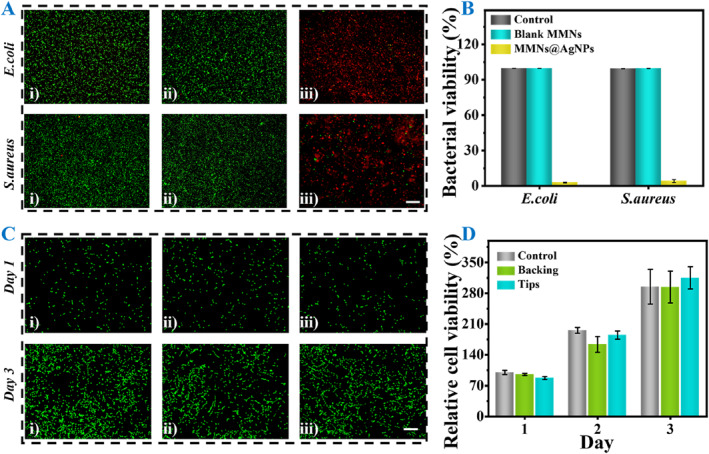
(A and B) Antibacterial effects of different microneedles. (i) Control, (ii) blank MMNs, and (iii) MMNs@AgNPs. Scale bar: 200 μm. (C) Fluorescence images of 3T3 cells in different groups. (i) Control, (ii) tips, and (iii) backing of MMNs. Scale bar: 200 μm. (D) Relative cell viability in different groups during 3 days. Relative to the OD value of cells in Control on day 1.

Taking advantage of the loaded AgNPs, MMN patches were imparted with outstanding antimicrobial capability. We co‐cultured the *Staphylococcus aureus* (*S. aureus*) and *Escherichia coli* (*E. coli*) with blank MMNs and AgNPs loading MMNs (MMNs@AgNPs) leaching, respectively. It has showcased that the MMNs@AgNPs group killed the highest number of both *E. coli* and *S. aureus* (Figure 3A), which exhibited the ability to inhibit bacterial growth. In comparison, bacteria in the control and blank MMNs groups were nearly unaffected. According to the statistic, the MMNs@AgNPs group displayed the lowest bacterial viability among the three groups (Figure 3B), demonstrating the effective antibacterial ability of the MMNs@AgNPs patches. To investigate the biotoxicity of such MMNs, both cytocompatibility and hemolysis evaluation were performed. Cytocompatibility was first assessed by co‐culturing GelMA tips and SilMA backing (SilMA@AgNPs) of MMNs with NIH/3T3 cells, respectively. As shown in Figure 3C, the morphology and growth rate of NIH/3T3 cells co‐cultured with backing (ii) and tips (iii) were both similar to those of the control group during 3 days. Furthermore, quantitative analysis of NIH/3T3 cell activity based on CCK‐8 demonstrated the satisfactory cytocompatibility (Figure 3D). Subsequently, hemolysis test was conducted co‐incubating GelMA tips and SilMA backing of MMNs with erythrocytes, respectively. The results exhibited neither backing nor tips had significant hemolytic effects (Supporting Information S1: Figure S4).

**FIGURE 4:**
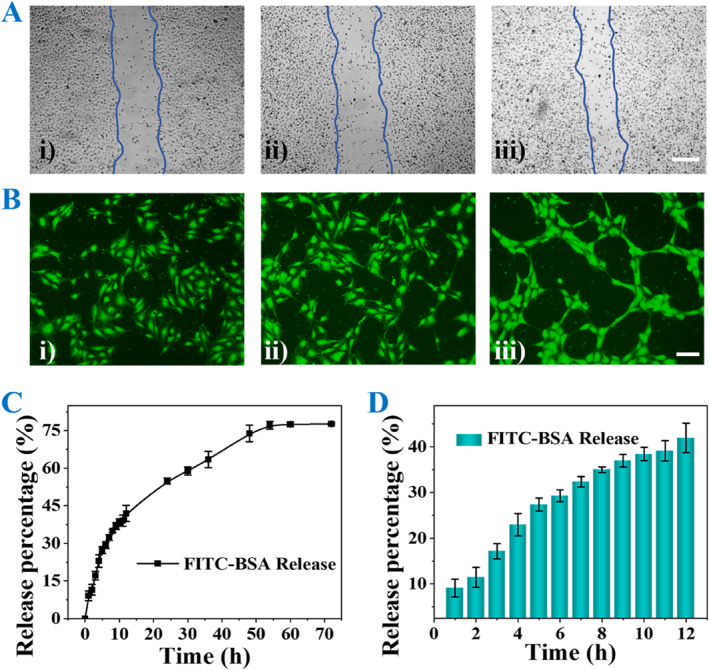
(A) Scratch test results in different groups for 24 h. (i) Control, (ii) blank MMNs, and (iii) MMNs@VEGF. Scale bar: 200 μm. (B) Tube formation in different groups. (i) Control, (ii) blank MMNs, and (iii) MMNs@VEGF. Scale bar: 50 μm. (C and D) Cumulative release of the FITC‐BSA from MMNs in (C) 72 h and (D) 12 h.

Vascular endothelial growth factor (VEGF) has been confirmed as a functional angiogenic factor. Therefore, MMNs encapsulating VEGF (MMNs@VEGF) were endowed with the ability to promote endothelial cell proliferation and vascular formation. To confirm the angiogenesis ability of MMNs@VEGF, human umbilical vein endothelial cells (HUVECs) were co‐cultured with the leaching liquid of blank MMNs and MMNs@VEGF, respectively. Scratch assay results showed the fastest scratch closure in MMNs@VEGF group within 24 h, indicating VEGF released from MMNs could accelerate cell proliferation (Figure 4A and Supporting Information S1: Figure S5A). Besides, tube formation results showcased the most ring structures in MMNs@VEGF group compared with the other two groups, suggesting VEGF released from MMNs could promote vascular formation (Figure 4B and Supporting Information S1: Figure S5B). Then, to investigate the release behavior of VEGF, FITC‐BSA with the same physicochemical properties acted as represent was encapsulated in MMNs. Based on the release profile curve, FITC‐BSA exhibited a relatively rapid release within 12 h (Figure 4D). In the following days, the release rate gradually decreased, reaching 77.6% over 72 h (Figure 4C).

**FIGURE 5:**
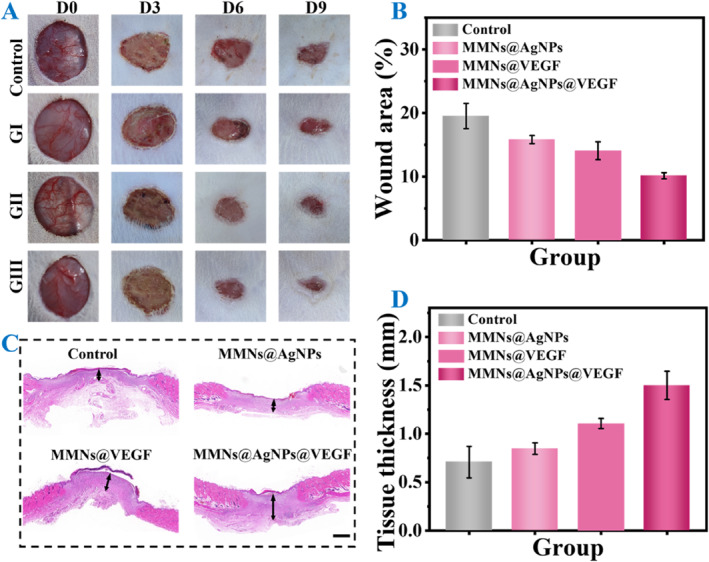
(A) Wound status in different groups. Control: PBS treated, GI: MMNs@AgNPs, GII: MMNs@VEGF and GIII: MMNs@AgNPs@VEGF. (B) Statistics of wound area in different groups on Day 9. (C) H&E staining of wounds in different groups on Day 9. Scale bars: 1 mm. (D) Tissue thickness of wounds on Day 9 in different groups.

To evaluate the in vivo therapeutic efficacy of MMNs, an infected wound rat model was established. Briefly, we created a circular wound with a diameter of 1.5 cm on rat dorsal skin, followed by *S. aureus* injection. Subsequently, rats were randomly divided into four groups: PBS treated (Control), MMNs with AgNPs treated (GI: MMNs@AgNPs), MMNs with VEGF treated (GII: MMNs@VEGF) and MMNs with AgNPs and VEGF treated (GIII: MMNs@AgNPs@VEGF). During the treatment days, we took photos of rats in each group to record the wound status and closure (Figure 5A). Compared to the Control group, wounds in GI, GII and GIII all exhibited different degrees of improvement. Among them, the wounds of GIII revealed the most effective closure, attributed to the multi‐function of MMNs, synergistically enhanced by the antibacterial effect of AgNPs and regeneration ability of VEGF. Quantitative results also demonstrated that the remaining wound area of GIII was the smallest (10.13%) compared with other groups (Figure 5B). Additionally, hematoxylin and eosin (H&E) staining of wound beds on Day 9 was conducted to further analyze the wound tissue (Figure 5C,D). The results showcased the thickest wound tissue in GIII, as confirmed by quantitative data.

**FIGURE 6:**
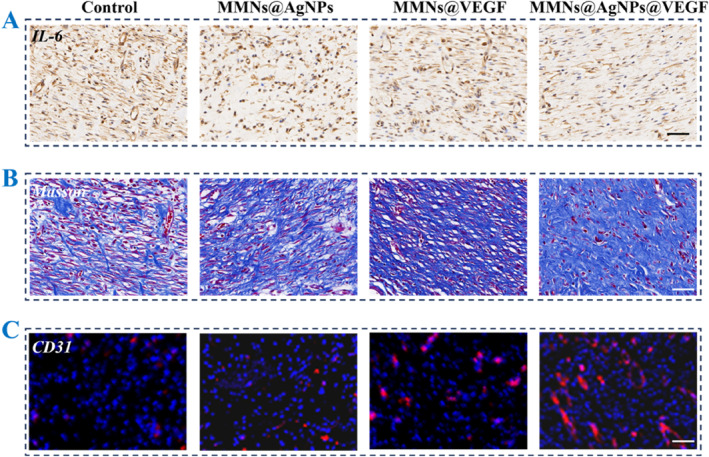
(A) IL‐6, (B) Masson staining, and (C) immunofluorescence staining of CD31 of rats wound beds treated with PBS (Control), MMNs@AgNPs, MMNs@VEGF and MMNs@AgNPs@VEGF on day 9.

To further evaluate the biological mechanism of treated wounds in each group, we performed (IL‐6) staining, Masson staining, and CD31 immunofluorescent staining. IL‐6 is an important inflammatory indicator and reflects the degree of wound inflammation, which was detected by IL‐6 staining. As shown in Figure 6A, the least positive area of IL‐6 was displayed in Group III compared to other groups, indicating the improved inflammation. Besides, collagen deposition, a key indicator in tissue regeneration, was assessed by Masson staining (Figure 6B). The results displayed the most collagen fiber formed and arranged in the wounds of G III, verifying the outstanding effect of tissue regeneration. In addition, angiogenesis is a crucial process in tissue remodeling, which provides nutrient transport within the wound microenvironment. We chose CD31 as a marker to evaluate the vascular situation in wounds (Figure 6C). Results revealed that wounds in GIII exhibited the most robust vascular formation and highest density, which can be attributed to the combination of functional angiogenic VEGF and antibacterial AgNPs. Taken together, such MMNs@AgNPs@VEGF can effectively accelerate the process of wound healing and tissue regeneration.

## 3 Conclusion

In sum, we developed a multifunctional microneedle patch that integrates different drugs into distinct regions for wound treatment. Such microneedle patches consisted of SilMA backing base loading AgNPs and GelMA tips containing VEGF, which were fabricated by the template replication. Importantly, the obtained microneedle patch exhibited excellent morphology and mechanical performance. In addition, the SilMA@AgNP backing demonstrated outstanding antimicrobial properties, which can act as an antimicrobial barrier against wound infections. Meanwhile, tips encapsulated with VEGF effectively promoted cell proliferation and angiogenesis, favorable for wound repair. Before in vivo experiments, we confirmed the satisfactory cytocompatibility and blood compatibility of such microneedle patches. Furthermore, rat experiments have demonstrated that such microneedle systems significantly promote wound healing and tissue regeneration.

## 4 Methods

### 4.1 Materials

2‐Hydroxy‐2‐methylpropiophenone (HMPP) was obtained from Aladdin Industrial Corporation in China. Fluorescein isothiocyanate‐bovine serum albumin (FITC‐BSA) was purchased from Bovogen. VEGF was purchased from Beyotime Corporation. *E. coli* and *S. aureus* were both obtained from BeNa Culture Collection. All reagents were of analytical grade and utilized as received.

### 4.2 Fabrication of MMN Patches

30% Gelatin methacryloyl (GelMA) pregel, 1% HMPP, and VEGF were mixed. This mixture was completely filled into the tip cavities of the negative mold. Then, the silk fibroin‐methacryloyl (SilMA) solution carrying Ag nanoparticles (AgNPs) was added to the mold as the backing. By UV solidification, two parts were combined. After drying, the MMN patches were finally demoulded. The optical microscope (Olympus) and scanning electron microscope (SEM) recorded the morphology of MMNs and the needle height.

### 4.3 Mechanical Performance of MMN Patches

A mechanical testing machine (Instron, USA) was employed to evaluate the mechanical performance of MMN patches. The patches were positioned tip‐up on a fixed platform. Then, the force sensor gradually closed toward the tips at a speed of 0.2 mm/s. The measurement was stopped until 500 μm.

### 4.4 Antibacterial Effect

For antibacterial testing, three groups were set up: Control, blank MMNs, and MMNs@AgNPs. Suspensions of *S. aureus* or *E. coli* (10^8^ CFU/mL) were added to each group and co‐incubated at 37°C, respectively. Afterward, the bacterial viability in each group was assessed using live/dead staining. The fluorescence images of *S. aureus* and *E. coli* were captured for each group.

### 4.5 Release Behaviors of Drugs

We chose FITC‐BSA as an alternative to VEGF due to its similar molecular weight. FITC‐BSA was encapsulated in GelMA hydrogel blocks. Then, the blocks were immersed in PBS solution containing collagenase. At time points, 100 μL of released medium was collected and replaced with fresh PBS. The release behavior of the drugs was assessed by measuring the fluorescence intensity. It was worth noting that the whole experiment was carried out under light‐protected conditions to prevent fluorescence quenching.

### 4.6 Scratch Assay

HUVECs (1 × 10^5^) were seeded in a 24‐well plate and cultured for 12 h. A scratch was then created using a pipette tip. Subsequently, three groups were established and co‐cultured with the HUVECs: the control group, the blank MMNs leachate group, and the MMNs@VEGF leachate group. After 24 h, cell growth in each group was observed and regarded under an optical microscope. Finally, the area of HUVECs proliferation was measured within 24 h.

### 4.7 Tube Formation Assay

100 μL of growth factor‐free Matrigel was added to each well of a 48‐well plate and then placed in 37°C incubator. Then, HUVECs (2 × 10^4^) were seeded in 48‐well plate and divided into three groups: the control group, the blank MMNs leachate group, and the MMNs@VEGF leachate group. HUVECs were co‐cultured with the leaching solution of the three groups. After 4 h, HUVECs in each group were stained with Calcein‐AM and recorded by fluorescence microscopy. Finally, the number of cell meshes of three groups was measured.

### 4.8 Cytocompatibility Experiment

Firstly, tips and backing of microneedles were exposed to UV light overnight, followed by immersed in DMEM culture medium for 24 h to prepare the leaching solution, respectively. Afterward, NIH 3T3 cells were cultured in the leaching solution for 3 days. Finally, NIH 3T3 cells in each group were stained with Calcein‐AM for each group on Day 1 and Day 3. Meanwhile, cell viability in each group was assessed by CCK‐8 on each day, respectively.

### 4.9 Hemocompatibility Experiment

Whole blood was first centrifuged and then resuspended. We set up four groups: the deionized water (DIW), PBS, MMNs tips, and backing groups. Then, erythrocytes were incubated in the leaching solution of four groups at 37°C for 1 h. Subsequently, the samples were centrifuged and photographed. Meanwhile, the OD values of the centrifuged supernatant from four groups were measured.

### 4.10 In Vivo Wound Healing Experiment

To establish an infected wound model, circular full‐thickness wounds with a diameter of 1.5 cm were conducted on rats dorsal skin, followed by *S. aureus* injection. Rats were randomly divided into four groups: PBS treated (Control), MMNs with AgNPs treated (GI: MMNs@AgNPs), MMNs with VEGF treated (GII: MMNs@VEGF) and MMNs with AgNPs and VEGF treated (GIII: MMNs@AgNPs@VEGF). Wound status was recorded every 3 days until 9 days of treatment. The wound areas were measured according to the images of wounds. After 9 days of treatment, all rats were euthanized, and wound tissues were collected for further analysis hematoxylin‐eosin (H&E) staining (IL‐6) staining, Masson staining, and CD31 immunofluorescent staining.

## Author Contributions

L.F. and L.W. contribute equally. H.Z., H.G. and M.L. conceived the idea and designed the experiment. L.F. and L.W. conducted experiments and wrote the manuscript. L.W. and X.W. revised the manuscript and checked the grammar.

## Ethics Statement

All animal experiments were approved by the Animal Investigation Ethics Committee of Wenzhou Institute of University of Chinese Academy of Sciences (No.WIUCAS23021603).

## Conflicts of Interest

Hongbo Zhang is an executive editor for *Smart Medicine* and was not involved in the editorial review or the decision to publish this article. All authors declare that there are no competing interests.

## Supporting information

Supporting Information S1
