# Supplementary material for: Multifunctional Silk and Gelatin Composed Microneedle Patches for Enhanced Wound Healing
Source: Smart Med. 2025 Feb 26;4(1):e137. doi: 10.1002/smmd.137 (PMC11862109; doi:10.1002/smmd.137)
Supplement: Supplementary file 1 — Supporting Information S1 [file SMMD-4-e137-s001.docx]

**Supporting Information**

**Multifunctional silk and gelatin composed microneedle patches for enhanced wound healing**

Lu Fan, Li Wang, Xiaoju Wang, Minli Li*, Hongcheng Gu*, Hongbo Zhang*

Dr. L. Fan, Dr. L. Wang, Dr. X. J. Wang, Prof. H. B. Zhang.

Pharmaceutical Sciences Laboratory, Åbo Akademi University, Turku 20520, Finland;

E-mail: Hongbo.Zhang@abo.fi

Prof. H. B. Zhang

Turku Bioscience Centre, University of Turku and Åbo Akademi University, Turku 20520, Finland

Dr. L. Fan, Dr. L. Wang, Dr. X. J. Wang, Prof. M. L. Li, Prof. H. C. Gu.

State Key Laboratory of Bioelectronics, School of Biological Science and Medical Engineering, Southeast University, Nanjing 210096, China

E-mail: [hcgu@seu.edu.cn;](mailto:hcgu@seu.edu.cn;) lml@seu.edu.cn


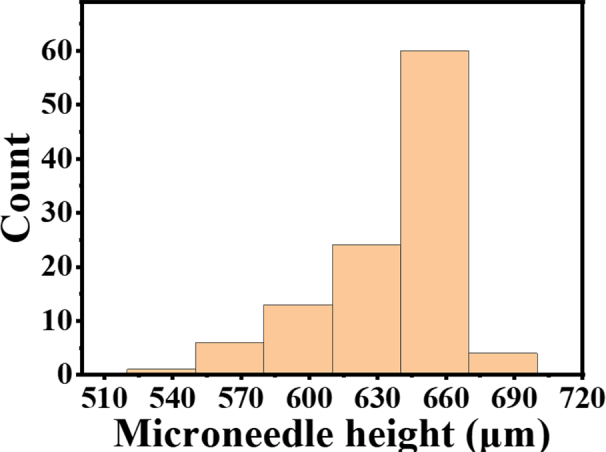


**Figure S1** Statistics of tip height of the MMNs


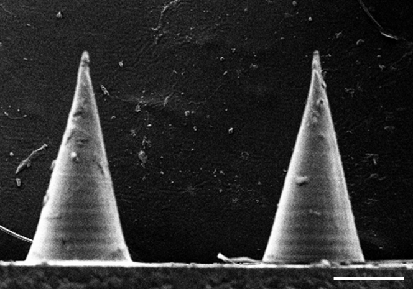


**Figure S2** SEM image of the multifunctional microneedle patch. Scale bar: 200 μm.


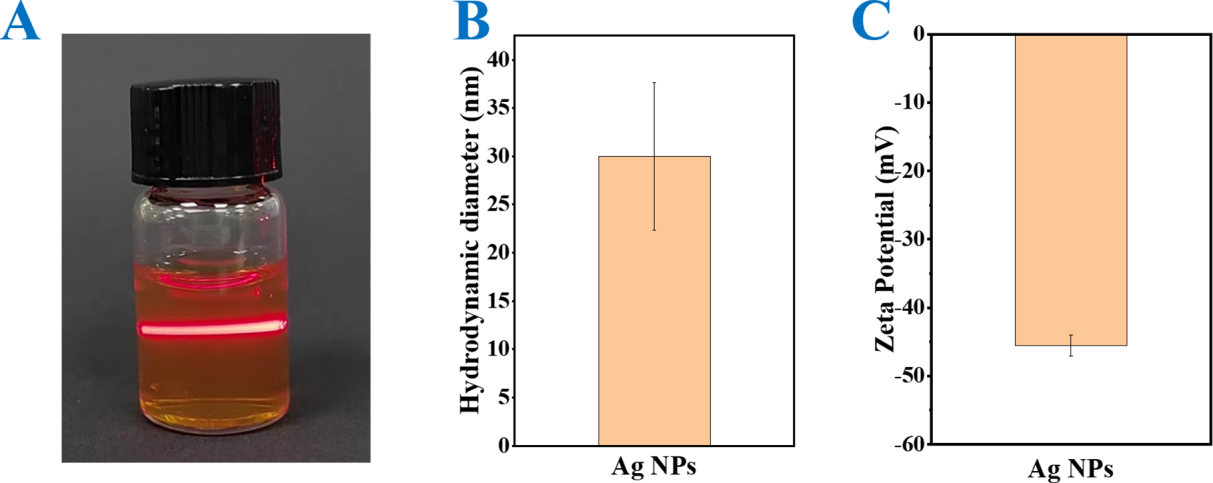


**Figure S3** (A) The Tyndall effect image, (B) hydrodynamic diameter and (C) zeta potential of AgNPs.


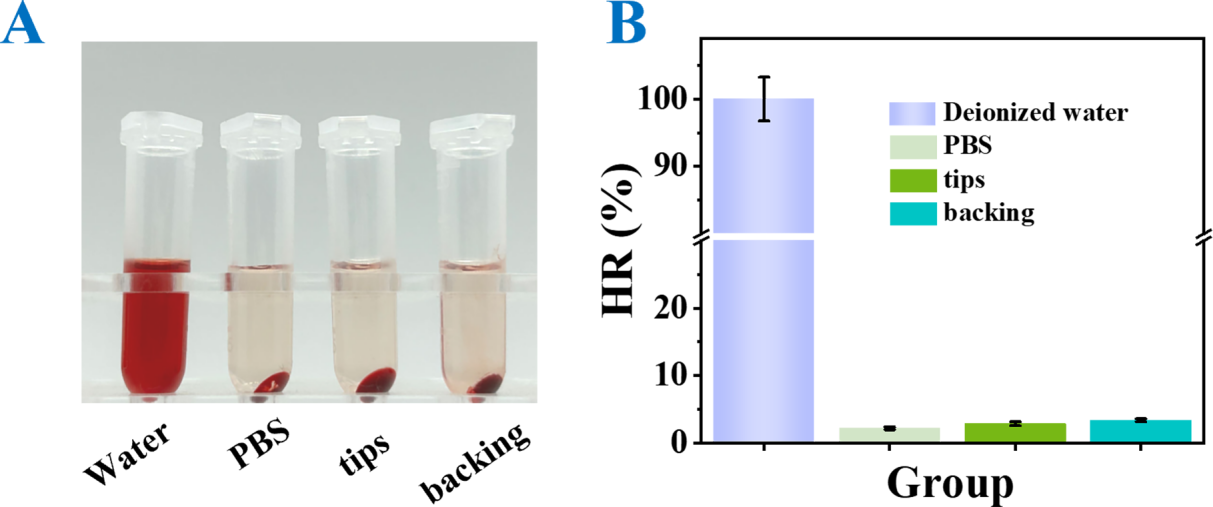


**Figure S4** Hemolysis test of tips and backing material of microneedle patches.


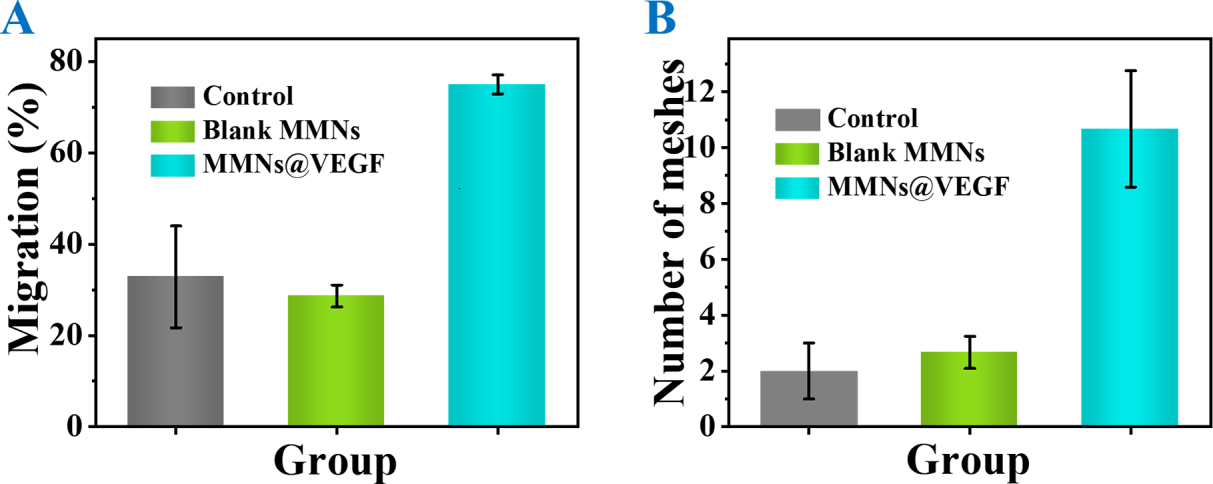


**Figure S5** (A) HUVECs migration in scratch test and (B) number of cell meshes in tube formation test of three groups.
